# Correction: Potential mechanisms and effects of AFB_1_-induced asthma: A comprehensive analysis based on network toxicology and molecular docking

**DOI:** 10.1371/journal.pone.0353138

**Published:** 2026-07-02

**Authors:** Zhiyue Yu, Ming Gao, Xiaowen Wu, Jiaxiang Li, Baijun Liu, Rui Wang, Jing Tan, Xiangyan Yu, Limei Geng

There is an error in affiliation 2 for author Limei Geng. The correct affiliation 2 is: Department of Respiratory Medicine, The Traditional Chinese Medical Hospital of Hebei Province, The First Affiliated Hospital of Hebei University of Traditional Chinese Medicine, Hebei University of Chinese Medicine, Shijiazhuang, Hebei, China.
