# Supplementary material for: Potential mechanisms and effects of AFB1-induced asthma: A comprehensive analysis based on network toxicology and molecular docking
Source: PLoS One. 2026 Jan 20;21(1):e0341172. doi: 10.1371/journal.pone.0341172 (PMC12818737; doi:10.1371/journal.pone.0341172)
Supplement: S2 File — This document provides detailed information on the versions of all databases and software used, along with extended methodological details and parameters for the molecular docking simulations. The datasets generated dring the current study are available in the zenodo respository, https://doi.org/10.5281/zenodo.17758079. (DOCX) [file pone.0341172.s002.docx]

| **Name** | **Versions** | **Dates** |
| --- | --- | --- |
| **ChEMBL** | ChEMBL_35 | 2025.1.31 |
| **STITCH** | v5.0 | 2025.1.31 |
| **SwissTargetPrediction** | For information: We have changed the look and feel of our tool. However, we have NOT changed the underlying technologies and parameters. Consequently, this updated Web tool provides exactly the same results as the previous version. | 2025.1.31 |
| **GeneCards** | v5.25.0 | 2025.1.31 |
| **OMIM** | Copyright (c) 1966-2025 Johns Hopkins University OMIM | 2025.2.1 |
| **TTD** | 2024 | 2025.2.1 |
|  | TTD: Therapeutic Target Database describing target druggability information. Nucleic Acids Research. 52(D1): 1465-1477 (2024). PMID: 37713619. |  |
| **STRING** | v11.5 | 2025.2.1 |
| **ProTox** | ProTox.3.0 | 2025.1.31 |
| **ADMETlab** | ADMETlab2.0 | 2025.1.31 |
| **R** | R version 4.4.1 | 2025.2.15 |
| **Packages** | ggvenn0.1.10 | 2025.2.15 |
| **Cytoscape** | Cytoscape_v3.10.3 | 2025.3.10 |
| **AutoDockVina** | AutoDockVina1.2.2 | 2025.2.17 |
| **PyMoL** | PyMoL2.5 | 2025.2.17 |

**Database and software versioning**

**Molecular Docking details**

**Table 1. Molecular docking parameters, results and validation of AFB1 with different proteins.**

| Target Protein | Ligand | Box Size (x,y,z) | Center (x,y,z) | Docking Score (kcal/mol) | Redocking Score (kcal/mol) | RMSD (Å) |
| --- | --- | --- | --- | --- | --- | --- |
| PTGS2 | AFB1 | 56.0, 62.0, 60.0 | 20.899, 37.499, 59.304 | -8.5 | -8.5 | 0.01 |
| ADRB2 | AFB1 | 40.0, 40.0, 40.0 | 31.902, 52.14, 45.377 | -8 | -8.1 | 0.001 |
| CYSLTR1 | AFB1 | 50.0, 40.0, 106.0 | 33.721, 22.14, 34.482 | -8.7 | -8.9 | 0.001 |
| PTGS1 | AFB1 | 68.0, 54.0, 58.0 | -36.709, -51.733, 2.080 | -7.7 | -7.7 | 1.8 |


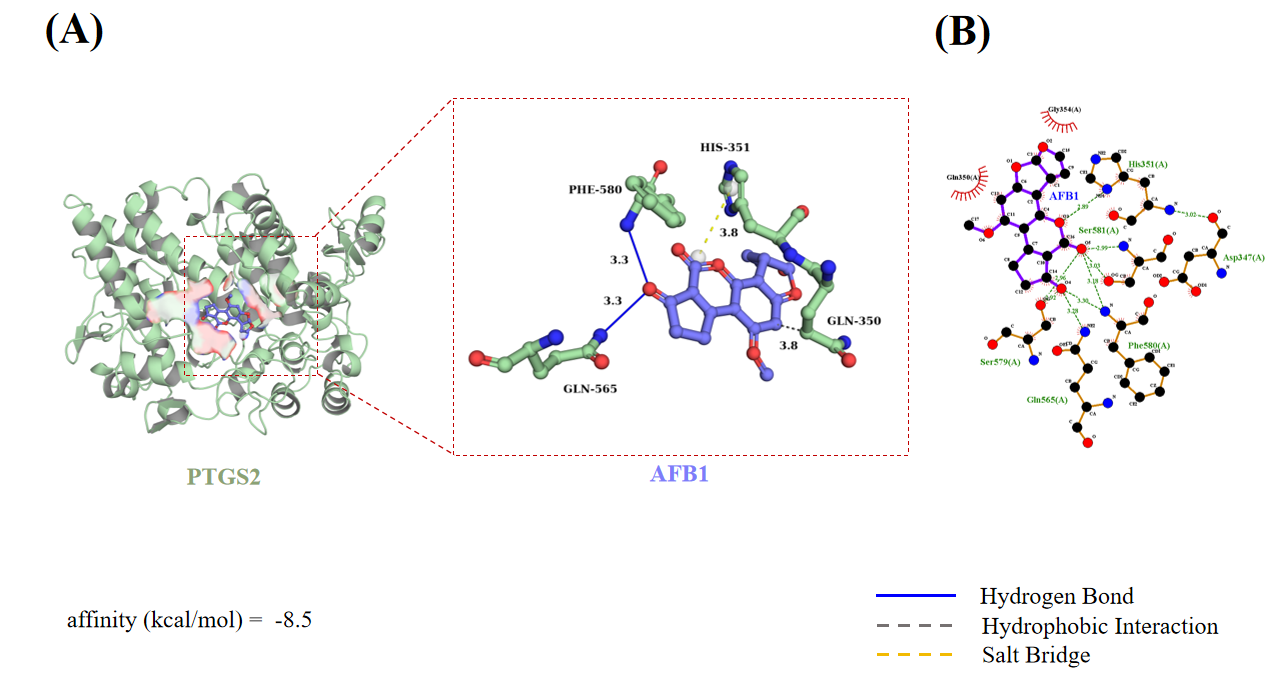


Fig5(A). Docking of AFB1 with its target PTGS1. The left cartoon represents the three-dimensional interactions of small molecule compound and target, and the right compound and its target in two-dimensional interactions.

As shown in Fig 5(A), purple represents AFB1 and green represents PTGS. The affinity score of AFB1 to the protein is -8.5 kcal/mol. The ligand and the target protein are stably bound through hydrophobic, hydrogen bonds, and salt bridges. 350A-GLN (3.78 Å) constitutes the hydrophobic interface; 565AGLN (2.50 Å) and 580A-PHE (2.36 Å) form crucial hydrogen bonds in the hydrogen bond network;, 351A-HIS forms a 3.85 Å salt bridge with the carboxyl group of the ligand, further enhancing the binding stability the complex.


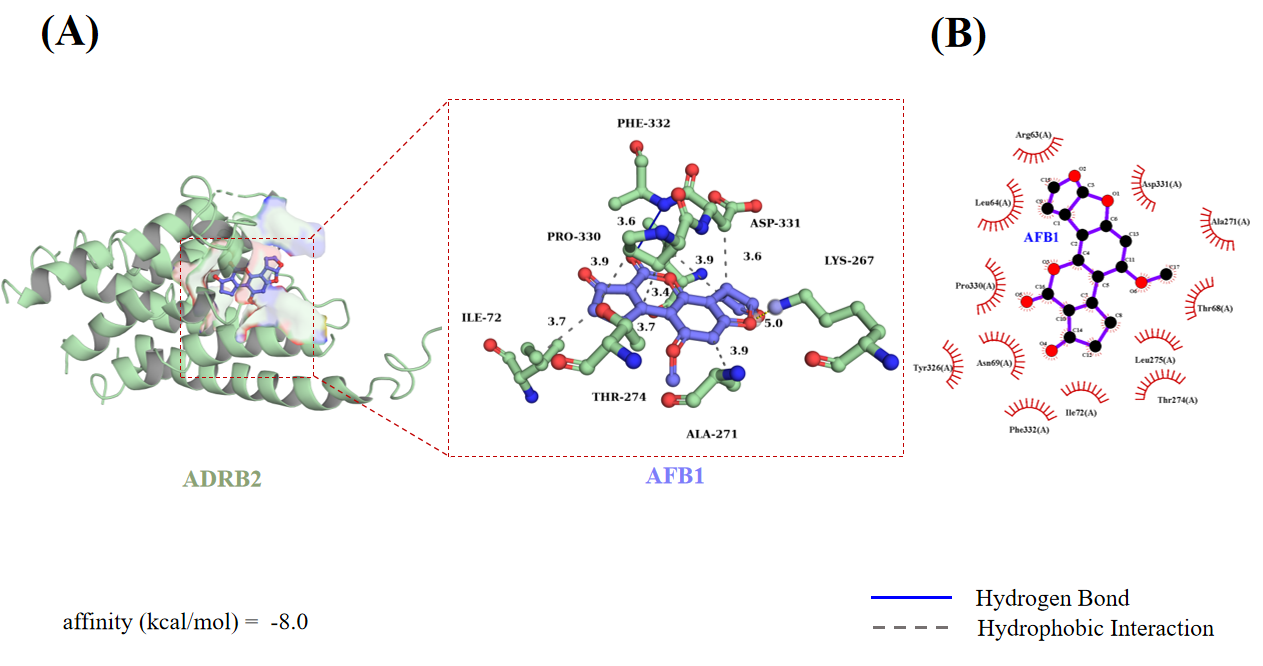


Fig5(B). Docking of AFB1 with its target ADRB2. The left cartoon represents the three-dimensional interactions of the molecule compound and its target, and the right compound and its target in two-dimensional interactions.

As shown in Fig5(B), where purple represents AFB1 and represents ADRB2. The affinity score of AFB1 to the protein was -8.0 kcal/mol. The ligand and the target protein were stably through hydrophobic interactions, hydrogen bonds and salt bridges. 64A-LEU (3.87 Å), 72A-ILE (372 Å), 271A-ALA (3.95 Å), 274A-THR (3.83 ), 330A-PRO (3.85/3.38 Å) and 331A-ASP (3.57 ) collectively constituted the hydrophobic interface; 332A-PHE (3.09 Å) formed a crucial hydrogen bond with the ligand; and267A-LYS formed a 5.00 Å salt bridge with the carboxyl group of the ligand, further enhancing the binding stability of the complex.


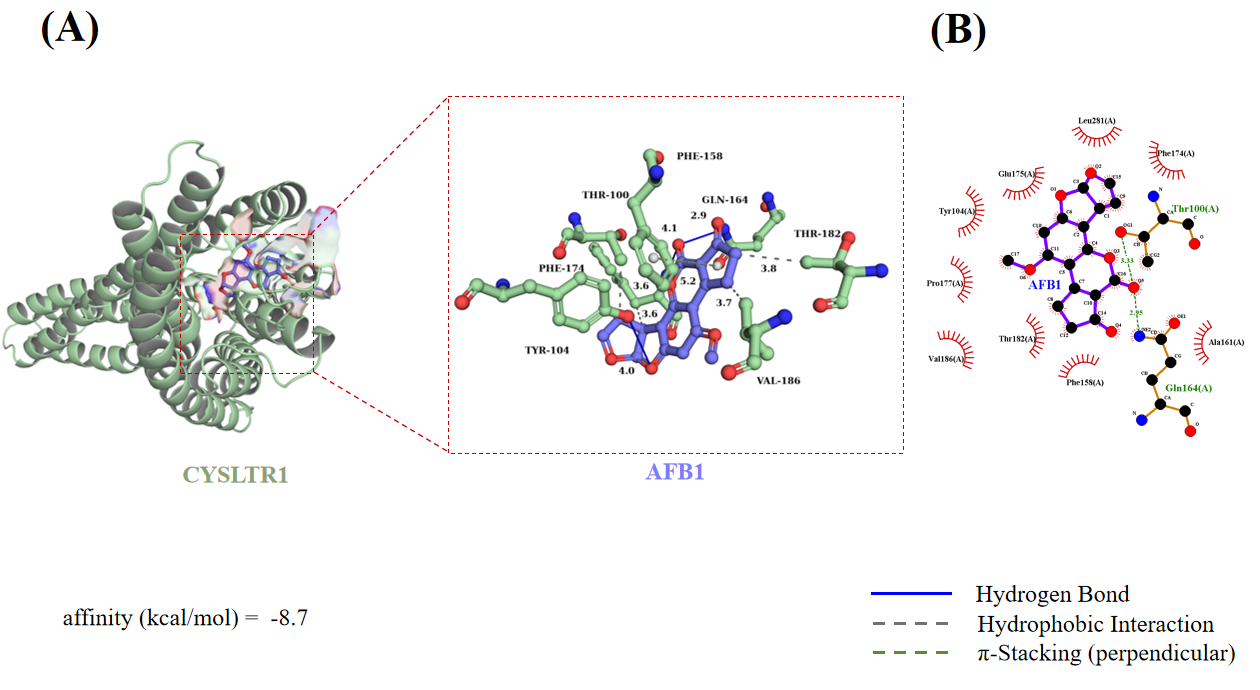


Fig5(C). Docking of AFB1 with its target CYSLTR1. The left cartoon represents the three-dimensional interactions the small molecule compound and its target, and the right compound and its target in two-dimensional interactions.

As shown in Fig5(C), where purple represents AFB and green represents CYSLTR1. The affinity score of AFB1 to the protein was -8.7 kcal/mol. The ligand and the target protein stably combined through hydrophobic interactions, hydrogen bonds and π-π stacking. The residues 100A-THR (3.62 Å) 174A-PHE (3.65 Å), 182A-THR (3.79 Å) and 16A-VAL (3.69 Å) constitute the hydrophobic interface; the hydrogen bond network, 104A-TYR (3.1 Å), 164A-GLN (2.06 Å) and 174A-PHE (3.28 Å) crucial hydrogen bonds with the ligand, among which the 2.06 Å ultra-short hydrogen bond of 164A-GLN is particularly crucial; the same time, 158A-PHE forms a T-shaped π-π stacking (5.22 Å) with the aromatic system of theand, further enhancing the binding stability of the complex.


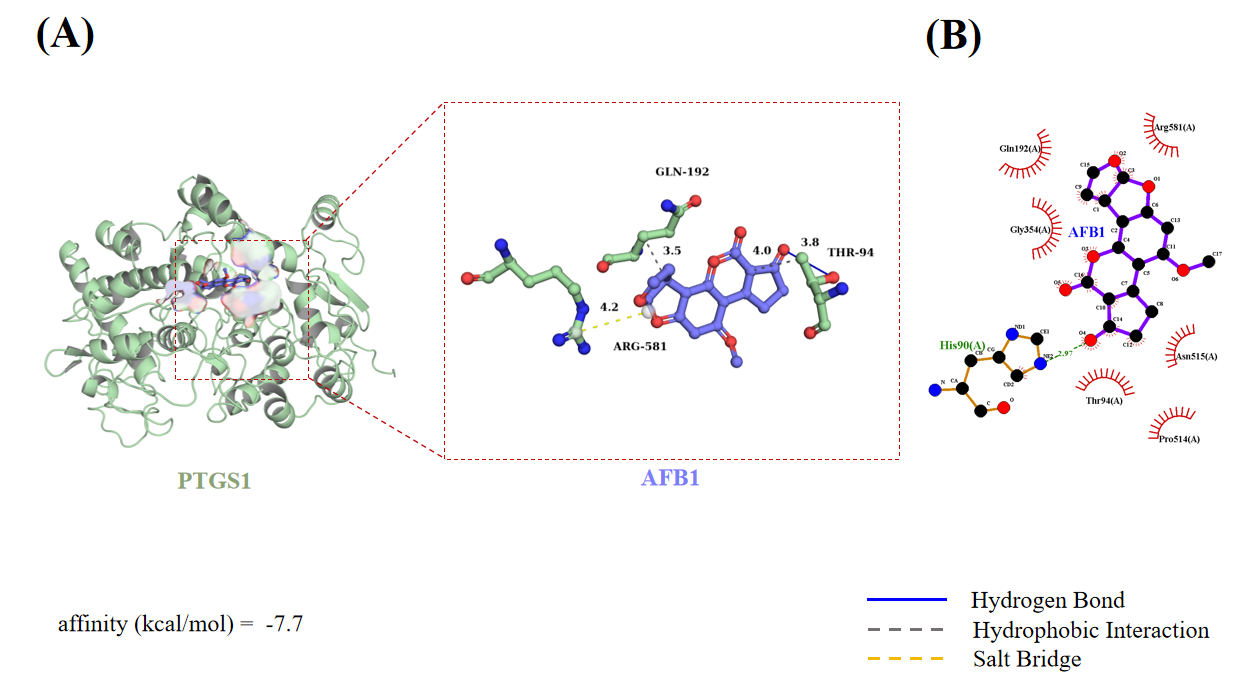


Fig5(D). Docking of AFB1 with its target PTGS1. The left cartoon represents the three-dimensional interaction of a small compound and its target, and the right compound and its target in two-dimensional interactions.

As shown in Fig5(D), where purple represents AFB1 and green represents PT1. The affinity score of AFB1 to the protein is -7.7 kcal/mol. The ligand and the target protein were stably bound through hydroph interactions, hydrogen bonds and salt bridges. 94A-THR (3.98 Å) and 192A-GLN (3.49Å) constitute the hydrophobic interface; 94A-THR (3.06 Å) forms a key hydrogen bond with the ligand; and 81A-ARG forms a 4.16 Å salt bridge with the carboxyl group of the ligand, further enhancing the binding stability of the complex

**Quantitative analysis table of molecular docking interactions**

| **Target Protein** | **Hydrogen Bonds** | **Hydrophobic Interactions** | **Salt Bridges** | **π-π Stacking** |
| --- | --- | --- | --- | --- |
| **PTGS2** | **Count:** 2 565A-GLN (2.50 Å) 580A-PHE (2.36 Å) | **Count:** 1 350A-GLN (3.78 Å) | **Count:** 1 351A-HIS (3.85 Å) | - |
| **ADRB2** | **Count:** 1 332A-PHE (3.09 Å) | **Count:** 6 64A-LEU (3.87 Å) 72A-ILE (3.72 Å) 271A-ALA (3.95 Å) 274A-THR (3.83 Å) 330A-PRO (3.85, 3.38 Å) 331A-ASP (3.57 Å) | **Count:** 1 267A-LYS (5.00 Å) | - |
| **CYSLTR1** | **Count:** 3 104A-TYR (3.11 Å) **164A-GLN (2.06 Å)** 174A-PHE (3.28 Å) | **Count:** 4 100A-THR (3.62 Å) 174A-PHE (3.65 Å) 182A-THR (3.79 Å) 186A-VAL (3.69 Å) | - | **Count:** 1 158A-PHE (5.22 Å) |
| **PTGS1** | **Count:** 1 94A-THR (3.06 Å) | **Count:** 2 94A-THR (3.98 Å) 192A-GLN (3.49 Å) | **Count:** 1 581A-ARG (4.16 Å) | - |
