# Supplementary material for: Potential mechanisms and effects of AFB1-induced asthma: A comprehensive analysis based on network toxicology and molecular docking
Source: PLoS One. 2026 Jan 20;21(1):e0341172. doi: 10.1371/journal.pone.0341172 (PMC12818737; doi:10.1371/journal.pone.0341172)
Supplement: Attachment — Submitted filename: Response to Reviewers.docx [file pone.0341172.s004.docx]

**Response to Reviewers**

We sincerely thank the editor and all reviewers for their valuable feedback that we have used to improve the quality of our manuscript. The reviewer comments are laid out below in *italicized font* and specific concerns have been numbered.Our response is given in blue font and changes to the manuscript are highlighted in yellow.

**Major comments:**

*Comments1: Database and software versioning: please provide detailed versions and access dates for all databases and tools used: ChEMBL, STITCH, SwissTargetPrediction, GeneCards, OMIM, TTD, STRING, ProTox, ADMETlab, and the specific software (R and packages, Cytoscape, AutoDock or equivalent).*

Response1:

Thank you for your valuable comments. We have thoroughly checked and supplemented the version information and access dates for all databases and software used in our study, as requested.

Please find the specific explanations below:

(1)**Version Information:** We have compiled the complete version details, access dates, and sources into a table in the Supplementary Materials/Methods section. For certain online databases and web tools that do not provide explicit version numbers (e.g., v1.2.3), we adopted the following alternative approaches to ensure traceability:

- For the **OMIM** database, we provided the copyright information displayed on its official page: "Copyright (c) 1966-2025 Johns Hopkins University."
- For the **TTD** database, we provided its most recent relevant citation: "Nucleic Acids Research. 52(D1): 1465-1477 (2024)", to indicate the data version it relies on.
- For tools that have been updated but whose core algorithms remain unchanged (e.g., **SwissTargetPrediction**), we cited their official statement: "...we have NOT changed the underlying technologies and parameters... provides exactly the same results as the previous version", to ensure the reproducibility of the results.

(2)**Access Dates:** The specific access dates for all online resources have been clearly listed in the table. This provides a precise timestamp for the state of the data at the time of retrieval.

We believe that providing official statements, citation literature, and precise access dates is sufficient to ensure the transparency of our methodology and the reproducibility of the results.

Thank you again for your careful review and constructive feedback.

The table is as follows.

| **Name** | **Versions** | **Dates** |
| --- | --- | --- |
| **ChEMBL** | ChEMBL_35 | 2025.1.31 |
| **STITCH** | v5.0 | 2025.1.31 |
| **SwissTargetPrediction** | For information: We have changed the look and feel of our tool. However, we have NOT changed the underlying technologies and parameters. Consequently, this updated Web tool provides exactly the same results as the previous version. | 2025.1.31 |
| **GeneCards** | v5.25.0 | 2025.1.31 |
| **OMIM** | Copyright (c) 1966-2025 Johns Hopkins University OMIM | 2025.2.1 |
| **TTD** | 2024 TTD: Therapeutic Target Database describing target druggability information. Nucleic Acids Research. 52(D1): 1465-1477 (2024). PMID: 37713619. | 2025.2.1 |
| **STRING** | v11.5 | 2025.2.1 |
| **ProTox** | ProTox.3.0 | 2025.1.31 |
| **ADMETlab** | ADMETlab2.0 | 2025.1.31 |
| **R** | R version 4.4.1 | 2025.2.15 |
| **Packages** | ggvenn0.1.10 | 2025.2.15 |
| **Cytoscape** | Cytoscape_v3.10.3 | 2025.3.10 |
| **AutoDockVina** | AutoDockVina 1.2.2 | 2025.2.17 |
| **PyMoL** | PyMOL 2.5 | 2025.2.17 |

*Comments2: GO/KEGG enrichment statistics: clearly state the multiple-testing correction method (e.g., Benjamini–Hochberg FDR), the p.adjust threshold used for significance, and attach full result tables (GO/KEGG term ID, description, geneRatio, p.adjust, and gene list) as supplementary CSV files.*

Response2:

Dear Reviewer, thank you for your comments. Regarding the statistical and visualization methods, we have added a corresponding description in the revised manuscript. Please refer to lines 111-113 (highlighted in yellow) in the document "Revised Manuscript with Track Changes". The added text is as follows:

“Statistical significance was corrected using the Benjamini-Hochberg FDR method, and we considered a P value below 0.05 as meaningful. We also used bubble plots to show the GO and KEGG results.”

Additionally, the relevant source data file has been deposited in the Zenodo open repository and is accessible via the following link and DOI: https://doi.org/10.5281/zenodo.17758079

*Comments3:* *STRING and PPI network parameters: Specify the confidence score cutoff, the evidence channels included (e.g., experiment, database, text mining), and the organism setting used. Please include the full STRING export (.tsv) as a supplementary file.*

Response3:

Thank you for raising this point. We would like to clarify that the key STRING parameters, including the organism and the minimum interaction score, were already specified in lines 100-101 of the Methods section in our original manuscript.

For your convenience, we summarize the full set of parameters used here:

**Organism:** Homo sapiens.

**Active Interaction Sources:** Textmining, Experiments, Databases, Co‑expression, Neighborhood, Gene Fusion, Co‑occurrence.

**Minimum Required Interaction Score:** 0.400 (Medium confidence)

**Query Proteins:** TACR1, MC4R, PDE5A, CHRM5, NR3C1, HRH1, CHRM2, ADRB1, BDKRB2, ADRB2, CXCR2, ADRB3, CYSLTR1, ADRA2C, CHRM4, CHRM3, PTAFR, ADORA1, PTGS2, TBXAS1, MMP9, CCR4, CCR5, PTGS1, MME, PIK3CD, ALOX5, ADORA2B, NOS2, MCL1, TRPV1.

Furthermore, as requested, the full STRING export in .tsv format has been deposited as supplementary data in the Zenodo open repository and is accessible via: https://doi.org/10.5281/zenodo.17758079

*Comments4:* *Hub gene identification: Indicate the algorithm and metric used in CytoHubba (e.g., Degree, MCC, Betweenness) and provide a table listing the top 10 genes with their centrality scores.*

Response4:

Thank you for your comment. We identified the hub genes using the Degree method in CytoHubba. The top 10 genes and their scores are listed in the table below.

Top 10 in network string_interactions_short.tsv ranked by Degree method

| **Rank** | **Name** | **Score** |
| --- | --- | --- |
| 1 | PTGS2 | 11 |
| 1 | ADRB2 | 11 |
| 3 | MMP9 | 10 |
| 4 | CYSLTR1 | 8 |
| 5 | PTGS1 | 7 |
| 6 | NR3C1 | 6 |
| 7 | TACR1 | 5 |
| 7 | TRPV1 | 5 |
| 7 | NOS2 | 5 |
| 7 | HRH1 | 5 |

Additionally, this table has been uploaded as a separate supplementary file (filename: degree_top_10) to the system for your review.

*Comments5:* *Molecular docking reproducibility: provide complete details on receptor and ligand preparation, grid box coordinates and size, exhaustiveness, number of poses, and validation steps (e.g., re-docking RMSD < 2 Å). Include a summary table with docking energy values (mean ± SD, kcal/mol), key interacting residues, and hydrogen/hydrophobic bonds. Add positive and negative controls if available.*

Response5:

In response to your suggestions, we have further refined the molecular docking section and optimized the corresponding figures. As the content is quite detailed, to maintain the conciseness and readability of the main text, we have moved the entire section to the zenodo under the title “docking” for your review.

Additionally, corresponding revisions have been made in the main text:

- Abstract (lines 17–19);
- Methods section (2.4, lines 115–127);
- Results section (3.4, lines 186–194).

All changes have been highlighted in yellow for your convenience.

We appreciate your guidance and support.

*Comments6:* *Data and code availability: deposit all raw data, scripts (e.g., Toxicity.venn.R), intermediate files (TXT, TSV, CSV), and figure sources in a public repository such as Zenodo or OSF and update the Data Availability Statement with a DOI link.*

Response6:

We thank the reviewer for the suggestion. As requested, we have added the 'Data Availability' statement to the main text (Lines 344-346). The added sentence, which reads "The datasets generated during the current study are available in the Zenodo repository, https://doi.org/10.5281/zenodo.17744835" has been highlighted in yellow for your convenience.

*Comments7:* *Discussion and limitations: the Discussion should go beyond result description. Please deepen biological interpretation, include literature comparisons, and explicitly acknowledge the in silico limitations and potential database biases. Rephrase causal statements into hypothesis-generating language (e.g., “may contribute,” “suggests”).*

Response7:

We sincerely thank the reviewer for this insightful suggestion. We have revised the Discussion section to deepen the biological interpretation, incorporate comparisons with existing literature, and explicitly acknowledge the limitations of our in silico approach and potential database biases. As advised, causal statements have been rephrased into hypothesis-generating language. All changes have been highlighted in yellow in the revised manuscript. We hope these revisions meet the journal's standards, and we welcome any further suggestions for improvement.

**Minor comments:**

*Comments1:* *Define all abbreviations at first mention (e.g., GPCR, ADMET).*

Response1:

We thank the reviewer for this valuable suggestion. We have systematically checked the manuscript and ensured that all abbreviations are defined at first mention. The corresponding revisions have been highlighted in yellow in the current version at the following lines: 69, 152, 156, 239, 260, 319, and 322. Should any further adjustments be needed, we would be pleased to make them accordingly.

*Comments2:* *Revise the writing for conciseness and grammar consistency; unify past tense throughout Methods and Results.*

Response2:

We thank the reviewer for this valuable suggestion. We have carefully proofread the manuscript to improve grammatical consistency and have unified the verb tense to the past tense throughout the Methods and Results sections. All revised areas have been highlighted in yellow for easy identification. We are committed to meeting the journal's standards and would be pleased to make any additional adjustments based on your further guidance.

*Comments3:* *Improve figure legends to include sample sizes, p.adjust scale, and database sources/versions.*

Response3:

We sincerely thank the reviewer for their valuable suggestions. We have comprehensively enhanced the content of all figure legends and made formatting adjustments to align with submission requirements. The revised figure files have been deposited in the Zenodo repository (https://doi.org/10.5281/zenodo.17744835) for your review. Detailed version information for the databases and tools used is available in the Supplementary Material under the section “Database and software versioning.” We trust that these revisions have fully addressed the comment raised and welcome any further feedback.

*Comments4:* *Include a table summarizing all databases and software (name, version, access date, URL).*

Response4:

We sincerely thank the reviewer for this valuable suggestion. As requested, detailed version information for all databases and tools used in this study has been compiled and is now available in the Supplementary Material under the section titled "Database and Software Versioning" for your review.

*Comments5:* Cite the official reference articles for the databases and tools (SwissTargetPrediction, STRING, ProTox-II, ADMETlab).

Response5:

We sincerely thank the reviewer for this suggestion. As requested, we have now cited the official reference articles for all the key databases and tools used in this study, including SwissTargetPrediction, STRING, ProTox-II, and ADMETlab. These citations have been added in lines 84–91 of the revised manuscript and are highlighted in yellow for your convenience.

*Comments6:* *Ensure that reference formatting follows PLOS ONE style (numbered citations in square brackets).*

Response5:

We thank the reviewer for highlighting this requirement. We have now reformatted all references throughout the manuscript using the "PLOS ONE" citation style in Zotero to ensure full compliance with the journal's formatting guidelines. Should any references still not fully meet the journal's requirements, we would be grateful for specific indications and will promptly make the necessary corrections.
