# Supplementary material for: Potential mechanisms and effects of AFB1-induced asthma: A comprehensive analysis based on network toxicology and molecular docking
Source: PLoS One. 2026 Jan 20;21(1):e0341172. doi: 10.1371/journal.pone.0341172 (PMC12818737; doi:10.1371/journal.pone.0341172)
Supplement: Attachment — Submitted filename: Response_to_Reviewers_auresp_2.docx [file pone.0341172.s005.docx]

**Response to Reviewers**

We sincerely thank the editor and all reviewers for their valuable feedback, which we have used to improve the quality of our manuscript. The reviewer comments are laid out below in *italicized font*. Our response is given in blue font and changes to the manuscript are highlighted in yellow.

*Comments1: Molecular docking methodology*

*The additional information provided in the supplementary material is very helpful. To improve reproducibility directly from the main text, it would be beneficial to briefly include: the exhaustiveness parameter used in AutoDock Vina, the number of poses generated, and whether any positive or negative controls were considered (or a short explanation if they were not used). A concise addition to the Methods would be sufficient.*

Response1:

We sincerely thank the reviewer for this excellent and practical suggestion to enhance methodological clarity. We have now added the requested details to the "Molecular Docking" section (Section 2.4) as follows:

“Molecular docking simulations were performed using AutoDock Vina 1.2.2 with the exhaustiveness parameter set to 32, generating nine binding poses for each ligand–target pair. The binding conformation with the lowest predicted binding energy was selected for subsequent analysis. No positive or negative control compounds were included in this study, as the primary aim was to comparatively evaluate the binding affinities and interaction patterns of the candidate drugs with their respective targets under identical docking conditions”.

This addition is found in lines 122-128 of the revised manuscript. We believe this makes our docking protocol fully transparent and reproducible. We are grateful for this suggestion and are happy to make further adjustments if needed.

*2. Interpretation of in silico findings*

*The authors have made an effort to adjust several statements in the Discussion, which is appreciated. For clarity, I kindly suggest slightly softening a few remaining phrases that may appear to imply causality based solely on in silico predictions. Emphasizing the exploratory and hypothesis-generating nature of network toxicology and docking will help align the interpretation with the strengths of these methods.*

Response2:

We are very grateful to the reviewer for this critical observation and guidance. We fully agree that the language must accurately reflect the predictive nature of our computational analyses.

We have therefore carefully reviewed the Discussion and Conclusion sections to soften statements that could be misinterpreted as implying direct causality. Key changes include replacing definitive terms with more tentative language to frame our findings as exploratory. These revisions are highlighted in yellow in lines 192-194, 209-211, 214, 239-240, 258, 326, 328, and 337-338.

We thank the reviewer for helping us improve the precision of our interpretation.

*3. Enrichment analysis presentation*

*The enrichment analysis is now clearer with the inclusion of FDR correction. If possible, adding metrics such as geneRatio or a similar indicator in the supplementary tables would give readers additional context regarding the weight of each enriched category.*

Response3:

We thank the reviewer for this helpful suggestion. Accordingly, we have added the **GeneRatio** metric to the enrichment analysis results. The updated supplementary tables containing this information have been uploaded with the resubmission. We appreciate this comment and are happy to make further changes if necessary.
